# Comparison of robotic, conventional, and endoscopic nipple-sparing mastectomy with immediate prosthetic breast reconstruction for breast cancer: A systematic review and meta-analysis

**DOI:** 10.17305/bb.2025.11687

**Published:** 2025-04-21

**Authors:** Na An, Wenjuan Wang, Dandan Dai, Fei Yuan, Yufeng Zhang

**Affiliations:** 1 Department of Breast Diseases, Weifang People’s Hospital, Weifang City, Shandong Province, China

**Keywords:** Breast cancer, robotic nipple-sparing mastectomy, RNSM, conventional nipple-sparing mastectomy, CNSM, endoscope-assisted nipple-sparing mastectomy, ENSM, network meta-analysis, NMA

## Abstract

In this network meta-analysis (NMA), we aimed to evaluate the relative efficacy of robotic nipple-sparing mastectomy (RNSM), conventional nipple-sparing mastectomy (CNSM), and endoscope-assisted nipple-sparing mastectomy (ENSM), each combined with immediate prosthetic breast reconstruction (IPBR), for the treatment of breast cancer. Relevant studies published up to June 15, 2024, were identified through searches of PubMed, Embase, the Cochrane Library, and Web of Science. Data extracted from these studies were analyzed using Stata 15.1 and the Gemtc 1.0.1 package in R 4.2.3. A Bayesian framework and a Markov Chain Monte Carlo model were employed to conduct the NMA. Additionally, a ranking chart was generated to compare the advantages and disadvantages of the surgical methods. Ten studies met the inclusion criteria and were included in the NMA. The results indicated that ENSM with immediate implant-based reconstruction was associated with a smaller incision compared to CNSM. RNSM combined with IPBR was linked to a lower incidence of total complications, Grade 3 complications, and nipple-areola complex necrosis than CNSM. Furthermore, RNSM with IPBR demonstrated a lower recurrence rate than CNSM. However, CNSM with IPBR showed better outcomes in terms of surgical time, hospital stay, and positive margin infiltration. In contrast, RNSM and ENSM, both combined with IPBR, outperformed CNSM in terms of incision length, complication rates, and recurrence outcomes.

## Introduction

Breast cancer is one of the most common malignant diseases, with a high incidence rate [1]. In 2020 alone, approximately 2.3 million new cases and 685,000 related deaths were reported [2]. The risk factors for breast cancer are multifactorial and include age, obesity, alcohol consumption, hormonal and reproductive factors, as well as genetic predispositions [3–5]. Despite the availability of various treatments, the five-year survival rate for metastatic breast cancer remains below 30% [6]. Due to its high heterogeneity, breast cancer requires tailored treatment strategies based on molecular subtypes [7]. Advances in molecular technology have enabled the classification of breast cancer into four distinct subtypes, facilitating earlier diagnosis and improving patient prognosis [8].

Treatment options for breast cancer vary depending on the stage, with the ultimate goal of prolonging life [9]. In 1894, radical mastectomy was introduced to ensure complete removal of pathological tissue while minimizing the risk of recurrence or metastasis [10]. Since then, mastectomy techniques have evolved to better preserve the breast’s natural appearance while maintaining oncological safety [11]. This evolution led to the development of new surgical approaches, including the introduction of nipple-sparing mastectomy (NSM) in the 1980s. NSM aims to improve esthetic outcomes and patient satisfaction by preserving the skin and nipple-areola complex (NAC). It enables safe cancer removal with local recurrence rates comparable to those of traditional mastectomies, but with higher patient satisfaction [12]. When paired with immediate breast reconstruction, NSM may reduce recurrence and mortality rates, minimize scarring, and further enhance patient satisfaction [13]. However, conventional NSM (CNSM) can result in a large, visible scar on the breast and carries a high risk of NAC necrosis [14].

Currently, endoscopic-assisted NSM (ENSM) and robotic-assisted NSM (RNSM) are emerging as new treatment trends, offering improved cosmetic outcomes and high patient acceptance [15]. One study reported that RNSM is associated with higher patient satisfaction, less blood loss, longer surgical times, and higher medical costs compared to ENSM [16]. Another study found that, relative to CNSM, RNSM involves longer surgical times and greater expense but results in a lower incidence of grade 2–3 breast complications [17]. In recent years, the number of patients undergoing minimal access breast surgery (MABS) has increased. Compared to conventional breast surgery, MABS effectively reduces scar length and shortens operative time, and is widely accepted by patients [18]. However, not all CNSM procedures require long or visible incisions. Recently, the inframammary approach has gained traction in NSM, particularly for its potential to optimize both aesthetic and functional outcomes [19].

Network meta-analysis (NMA) is a statistical method used to compare the efficacy of different treatments, including those lacking direct comparisons [20, 21]. The core principle of meta-analysis is to statistically combine results from multiple independent studies on the same topic to draw a more robust conclusion. ENSM or RNSM offers esthetic benefits—such as a scar-free procedure and improved patient satisfaction—but these techniques are often associated with longer operative times and higher costs [15, 22]. Currently, few studies have directly compared the effectiveness of various NSM approaches combined with immediate breast reconstruction. Therefore, in this NMA, we aimed to evaluate the relative efficacy of RNSM, CNSM, and ENSM, each combined with immediate prosthetic breast reconstruction (IPBR) in the treatment of breast cancer.

## Materials and methods

### Search strategy

Our systematic review and meta-analysis was conducted according to the Preferred Reporting Items for Systematic Reviews and Meta-Analyses (PRISMA) guidelines [21, 23]. The protocol has been registered in the Open Science Framework (OSF) registry with the registration code osf.io/5j3dk. We searched for relevant articles up to June 15, 2024, in the Embase, PubMed, Web of Science, and Cochrane Library databases. All searches used medical subject headings and common keywords, including “Endoscopy,” “Endoscopic,” “Robotic,” “Robotics,” “Robot,” “Robots,” “Robotically,” “Breast,” “Mammary,” “Neoplasm,” “Neoplasms,” “Tumor,” “Tumors,” “Cancer,” “Cancers,” “Carcinoma,” “Carcinomas,” “Implantation,” “Implantations,” “Reconstruction,” “Reconstructions,” “Flap,” “Flaps,” “Reconstructive,” “Mammaplasty,” “Mammaplasties,” “Mammoplasty,” “Mammoplasties,” “Mastectomy,” “Mastectomies,” “Mammectomy,” “Mammectomies.” The key terms based on the PICOS search method are presented in Table 1 [24]. We did not limit the outcomes or study design in the search terms to avoid missing potentially relevant studies from our review. The detailed search syntax and the number of records retrieved from each database are shown in Table S1. The literature was imported into EndNote X20 and initially screened by reading the titles and abstracts. Subsequently, the full texts were reviewed to exclude studies that did not meet the inclusion criteria. The remaining studies were included in the final analysis.

**Table 1:** PICOS framework for key search terms

**Category**	**Search terms**
Population	“Breast Neoplasm” OR “Breast Tumor” OR “Breast Cancer” OR “Breast Carcinoma”
Intervention	Endoscopic-assisted nipple sparing mastectomy
Comparision	“Robotic-assisted nipple sparing mastectomy” OR “Conventional nipple sparing mastectomy”
Outcomes	Incision length (not limited in search terms)
Study design	Clinical trials and observational studies (not limited in search terms)

### Inclusion and exclusion criteria

The inclusion criteria for this study were as follows:

1. **Participants**: Patients with breast cancer undergoing RNSM, CNSM, ENSM, and IPBR.
2. **Intervention**: Studies on RNSM combined with immediate breast reconstruction, ENSM combined with immediate breast reconstruction, and traditional surgery combined with immediate breast reconstruction.
3. **Outcomes**: (a) Incision length (cm); (b) Total operation time (min); (c) Blood loss (mL); (d) Hospital stay (days); (e) Overall complication rate and incidence of grade 3 complications (Clavien–Dindo classification), as well as specific complication rates; (f) Positive margin involvement, where cancer cells are found at the edge of the removed tumor tissue, indicating incomplete tumor removal and possible remaining cancer cells in the patient’s body; (g) Recurrence. type is clinical trial or observational study.
4. **Study type**: Clinical trials or observational studies.

The exclusion criteria for this analysis included: animal studies; reviews, meta-analyses, case reports, conference abstracts, editorials, trial registrations, guidelines, books, and notes; studies with inconsistent themes; non-English publications; and retracted articles.

### Data extraction

Initially, studies were screened according to the predefined inclusion and exclusion criteria. Data extracted from the eligible publications included the first author, publication year, study design, study duration, and sample size. Patient characteristics were also collected, such as age, body mass index (BMI), lymph node surgery, tumor size, tumor stage, and histopathological grade. Two researchers independently performed data extraction, and any discrepancies were resolved through consultation with a third researcher.

**Figure 1.**
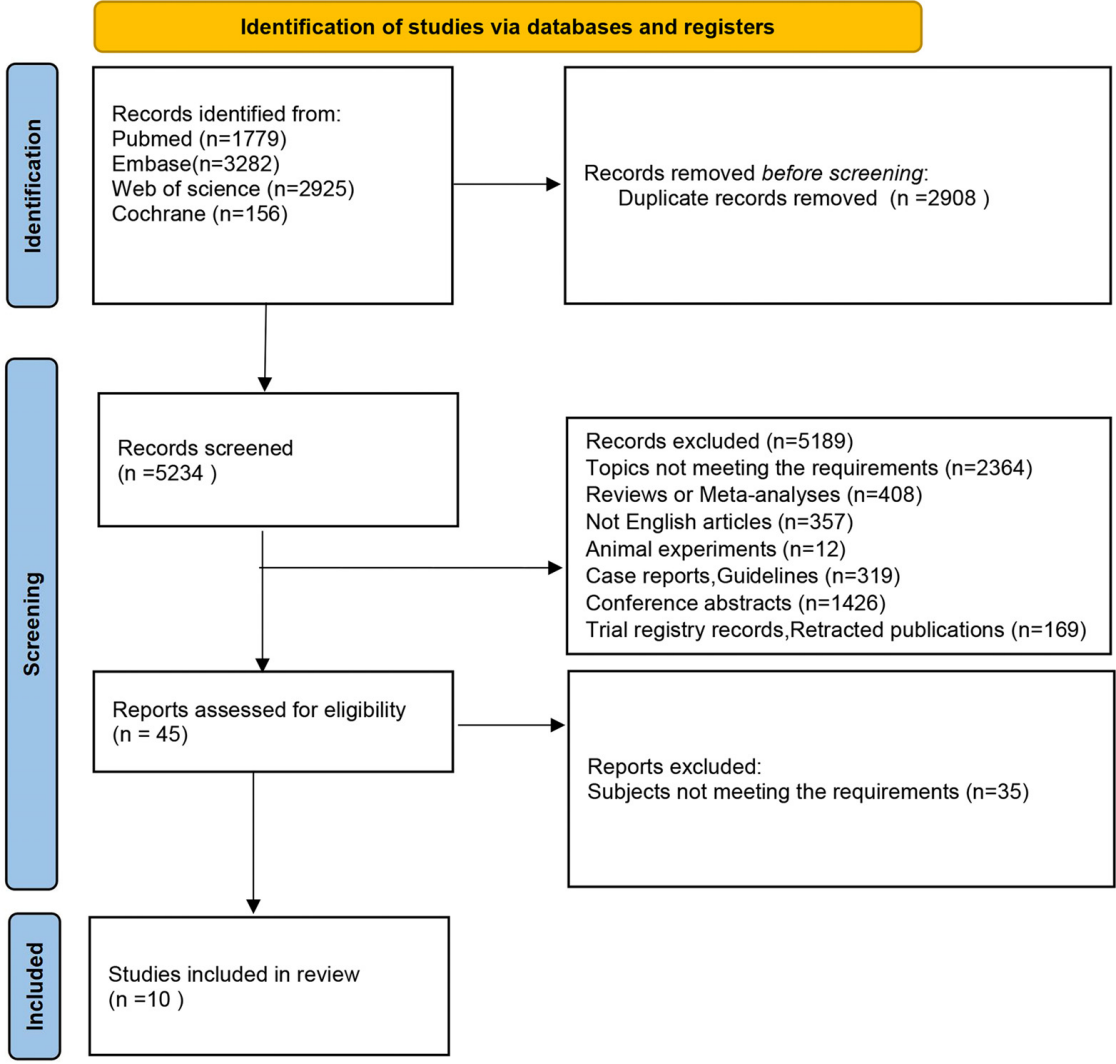
Flowchart of the search process for the network meta-analysis.

### Literature quality assessment

Randomized controlled trials were assessed using the modified Jadad scale [25], which evaluates random sequence generation, allocation concealment, blinding, and reporting of dropouts and losses to follow-up. Studies scoring 1–3 points were considered low quality, while those scoring 4–7 points were classified as high quality. Cohort and case-control studies were evaluated using the Newcastle–Ottawa Scale (NOS) [26], which scores studies on a scale from 0–9 points: 0–3 points indicate low quality, 4–6 points medium quality, and 7–9 points high quality. Non-randomized controlled intervention studies were assessed using the Methodological Index for Non-Randomized Studies (MINORS) [27], which consists of 12 items with a maximum total score of 24 points.

### Meta-analysis

This study performed a NMA using a Bayesian framework and a Markov Chain Monte Carlo (MCMC) model. The analysis was conducted with four chains, an initial burn-in of 20,000 iterations, followed by 50,000 sampling iterations, with a step size of one.

### Statistical analysis

Data analysis was conducted using the Gemtc 1.0.1 package in R (version 4.2.3; R Foundation for Statistical Computing, Vienna, Austria) and Stata software (version 15.1; StataCorp, College Station, TX, USA). Heterogeneity was assessed using the *I*^2^ statistic [28]. Model consistency was evaluated by comparing the Deviance Information Criterion (DIC) values of the consistency and inconsistency models, with a smaller DIC indicating a better model fit. A DIC difference of less than five was considered indicative of acceptable model consistency [29]. For continuous outcomes, such as incision length, total operation time, blood loss, and hospital stay, weighted mean differences (WMDs) with 95% confidence intervals (CIs) were calculated [30]. For binary outcomes—including complication rate, positive margin involvement, and recurrence—relative risks (RRs) with 95% CIs were reported [24]. Forest plots were used to display both direct and indirect comparisons of RRs or WMDs with their respective 95% CIs. Additionally, a ranking plot was generated to visualize the comparative advantages and disadvantages of each surgical approach.

## Results

### Inclusion of literature

Based on the search strategy, a total of 8142 articles were initially retrieved. All records were imported into EndNote X20 for screening. After the removal of duplicates and exclusion of irrelevant titles and abstracts, 45 articles remained. Of these, 35 were excluded for not meeting the inclusion criteria, resulting in 10 studies being included in the final meta-analysis [16, 17, 31–38]. A detailed flowchart outlining the literature screening process is shown in Figure 1.

### Quality evaluation

The meta-analysis included a total of 1525 patients, with 504 in the RNSM group, 771 in the CNSM group, and 250 in the ENSM group. Detailed baseline characteristics—including age, BMI, tumor size (cm), lymph node surgery, TNM stage, histopathological grade, and follow-up duration (months)—are summarized in Table 2. The quality assessment of the included studies is provided in Tables S2–S5. According to the NOS, the studies scored between six and eight points, indicating an overall moderate to high methodological quality.

**Table 2:** Baseline information

	**Country**	**Group**	***N***	**Age, year**	**BMI, kg/m^2^**	**Tumor size (cm)**	**Lymph node surgery**	**TNM stage**	**Histopathologic grade**	**Follow-up, months**
Houvenaeghel, 2021	France	RNSM	87	Mean, 47.8	≤24.9, 73; 25–29.9, 9; ≥30, 5	NR	NR	NR	NR	12
		CNSM	142	Mean, 52.7	≤24.9, 119; 25–29.9, 17; ≥30, 6	NR	NR	NR	NR	12
Lai^a^, 2020	Taiwan, China	RNSM	40	49 ± 10*	NR	2.5 ± 2.5*	SLNB only, 31; SLNB then ALND, 7; ALND, 1; Not down, 1	0, 9; I, 11; IIa, 11; IIb, 6; IIIa, 2; IIIc, 1	I, 8; II, 17; III, 6	13.5 ± 6.8*
		ENSM	91	49 ± 10*	NR	2.2 ± 1.5*	SLNB only, 75; SLNB then ALND, 13; ALND, 1; Not down, 2	0, 28; I, 28; IIa, 23; IIb, 8; IIIa, 4; IIIc, 0	I, 13; II, 54; III, 16	45.6 ± 25.5*
Lai^b^, 2020	Taiwan, China	RNSM	54	48 ± 9.3*	NR	2.5 ± 2.3*	SLNB only, 40; SLNB then ALND, 11; ALND, 2; Not down, 1	0, 8; I, 14; IIa, 16; IIb, 7; IIIa, 6	I, 13; II, 25; III, 9	14.6 ± 8.8*
		CNSM	62	49 ± 11*	NR	2.5 ± 1.6*	SLNB only, 37; SLNB then ALND, 12; ALND, 6; Not down, 7	0, 14; I, 15; IIa, 17; IIb, 7; IIIa, 2	I, 8; II, 34; III, 11	47.3 ± 19.6*
Lai, 2024	Taiwan, China	CNSM	73	46.1 ± 8.0*	<18, 4; 18–24, 45; ≥24, 24	NR	No, 9; SLNB only, 48; SLNB then ALND, 9; ALND, 7	NR	NA, 16; I, 14; II, 34; III, 9	25.5 ± 8.5*
		ENSM	84	46.9 ± 8.3*	<18, 4; 18–24, 54; ≥24, 26	NR	No, 5; SLNB only, 62; SLNB then ALND, 12; ALND, 5	NR	NA, 9; I, 17; II, 36; III, 22	26.9 ± 6.9*
		RNSM	76	48.2 ± 9.5*	<18, 3; 18–24, 59; ≥24, 14	NR	No, 13; SLNB only, 48; SLNB then ALND, 6; ALND, 9	NR	NA, 16; I, 10; II, 33; III, 17	28.4 ± 8*
Lee, 2021	Korea	ENSM	20	47.2 ± 9.5*	24.1 ± 3.8*	NR	ALND, 2	0, 10; Ia,3; IIa, 5; IIb, 1; IIIa, 1	NR	NR
		CNSM	25	44.6 ± 9.6*	22.3 ± 3.6*	NR	ALND, 4	0, 6; Ia, 11; IIa, 5; IIb, 2; IIIa, 1	NR	NR
Moon, 2021	Korea	RNSM	40	46 ± 8*	22.2 ± 3.5*	1.6 ± 1.3	SLNB only, 37; SLNB then ALND, 3	NR	Grade I, 13; Grade II, 23; Grade III, 4	NR
		CNSM	41	49 ± 10*	23.9 ± 3.6*	1.8 ± 1.1	SLNB only, 36; SLNB then ALND, 5	NR	Grade I, 10; Grade II, 21; Grade III, 9	NR
Park, 2022	Korea	RNSM	167	45 (28–71)ˆ	<25, 152; ≥25, 15	NR	NR	≤Stage I, 111; >Stage I, 45; Benign, 11	NR	18
		CNSM	334	44 (23–71)ˆ	<25, 294; ≥25, 40	NR	NR	≤Stage I, 227; >Stage I, 85; Benign, 22	NR	
Toesca, 2022	Italy	CNSM	40	45.5 (29–62)ˆ	Underweight, 8; Normal weight (18.5–24.9 kg/m2)ˆ, 32	NR	NR	0, 5; Ia, 15; IIa, 9; IIb, 6; IIIa, 0; IV, 0	NR	28.6 (range 3.7–43.3)
		RNSM	40	44.5 (30–60)ˆ	Underweight, 4; Normal weight (18.5–24.9 kg/m2)ˆ, 36	NR	NR	0, 7; Ia, 12; IIa, 9; IIb, 3; IIIa, 2; IV, 1	NR	
Wang, 2023	China	ENSM	38	42.00 (36.75–51.75)ˆ	21.91 (19.98–24.10)ˆ	NR	NR	NR	NR	51.5
		CNSM	26	45.50 (39.00–59.00)ˆ	25.57 (21.11–28.10)ˆ	NR	NR	NR	NR	
Qiu, 2022	China	ENSM	17	35.9 ± 6.4*	21.3 ± 1.3*	NR	SLNB, 12; ALND, 5	NR	NR	NR
		CNSM	28	39.1 ± 7.7*	22.3 ± 4.6*	NR	SLNB, 13; ALND, 15	NR	NR	NR

Note: *Mean ± SD; ˆMedian (Q1,Q3). NR: Non-reported; RNSM: Robotic nipple sparing mastectomy; CNSM: Conventional NSM; ENSM: Endoscopic-assisted NSM; SLNB: Sentinel lymph node biopsy; ALND: Axillary lymph node dissection; TNM: Tumor, Node, Metastasis.

### Results of meta-analysis

#### Incision length (cm)

**Figure 2.**
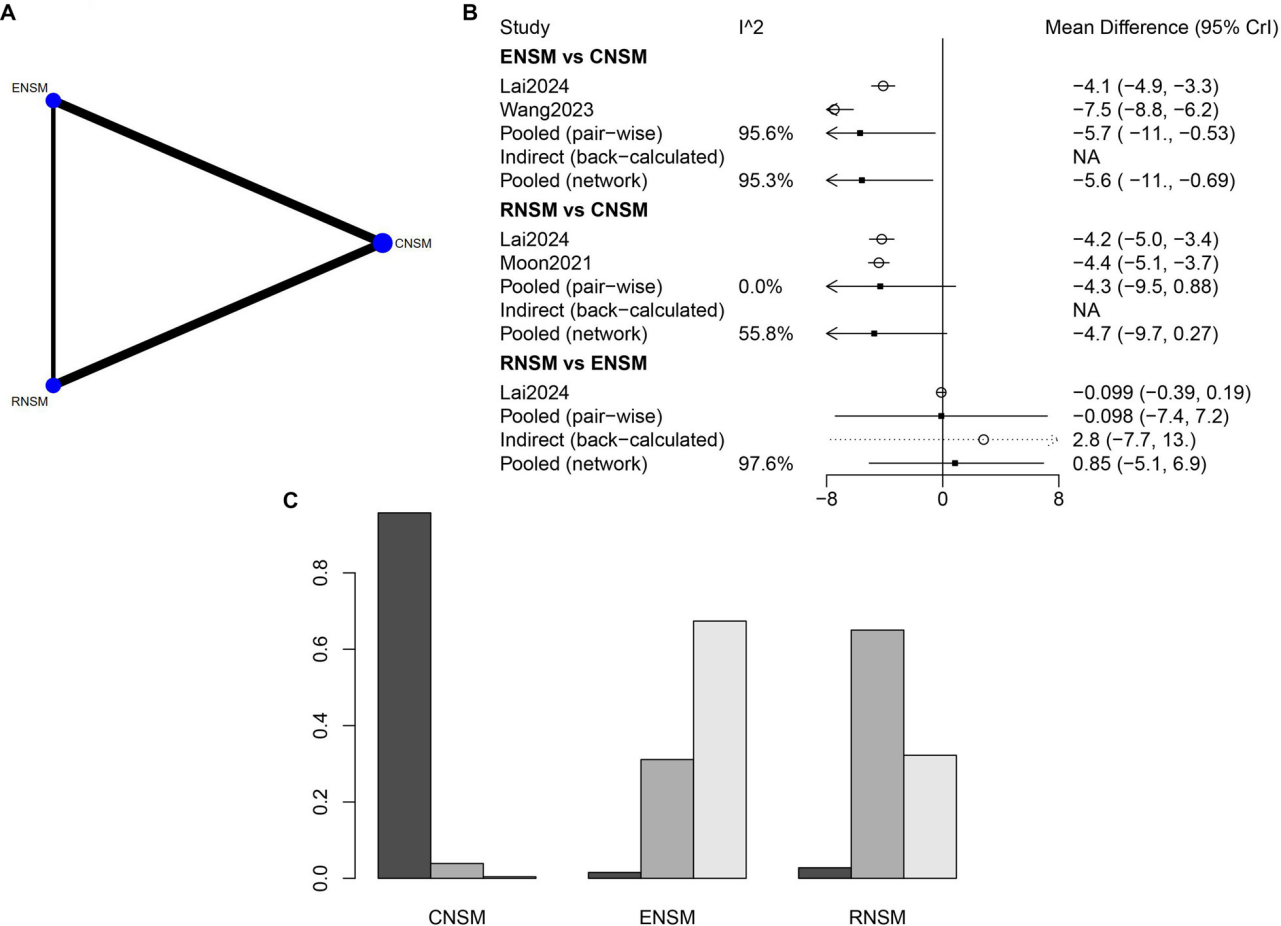
**Meta-analysis results of incision length.** (A) Network diagram; (B) Forest plot; (C) Sorting probability graph.

The connections between RNSM and CNSM, as well as between ENSM and CNSM, reflect a greater number of direct comparison studies involving CNSM, suggesting a larger sample size for this group (Figure 2A). The incision length was significantly shorter in the ENSM group compared to the CNSM group, with a WMD of −5.57 (95% CI: −10.74 to −0.69). No significant differences in incision length were observed between the other groups (Figure 2B). Overall, ENSM appeared to be the most favorable surgical approach in terms of minimizing incision length, followed by RNSM and then CNSM (Figure 2C).

#### Total operation time (min)

**Figure 3.**
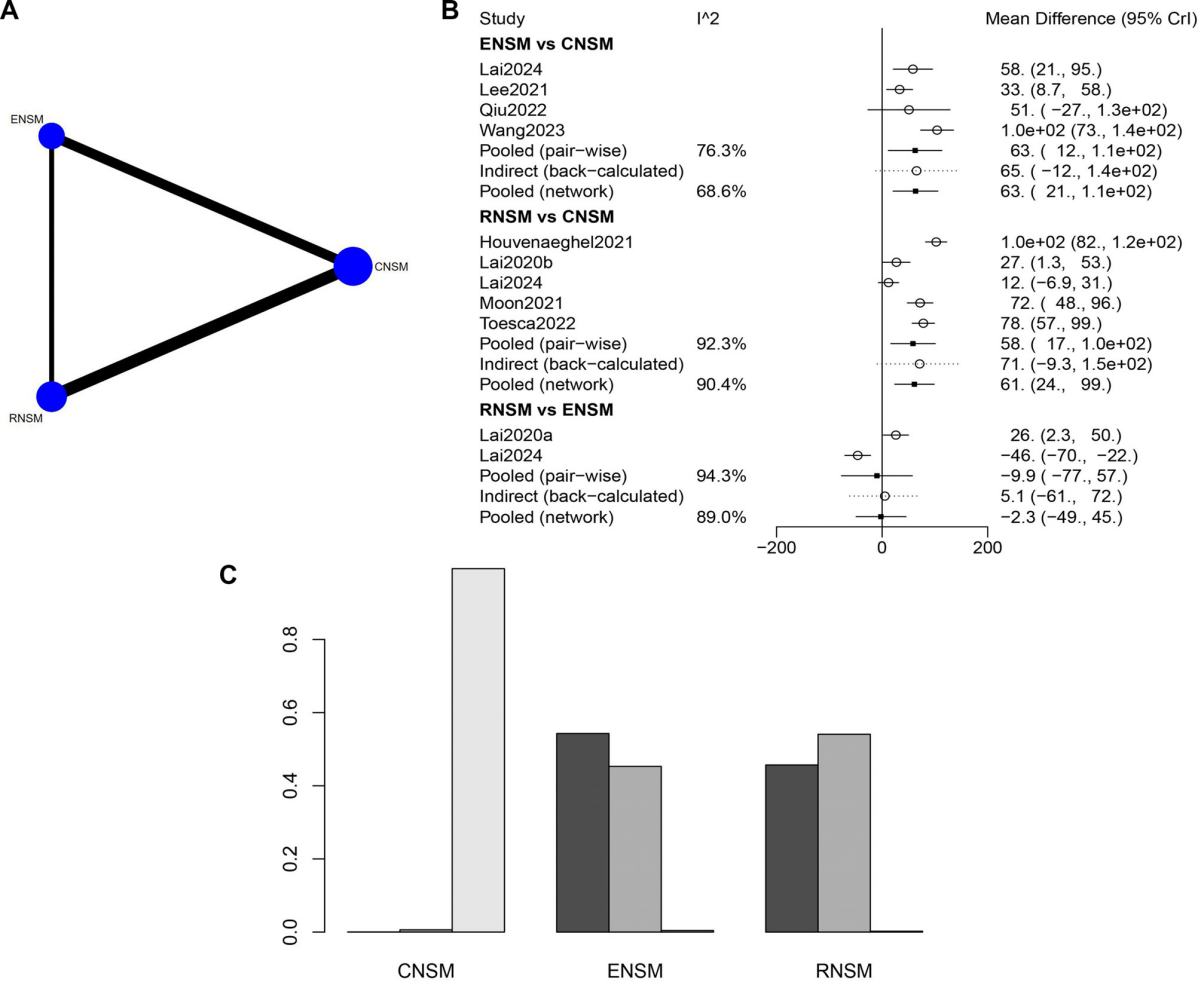
**Meta-analysis results of all operation time.** (A) Network diagram; (B) Forest plot; (C) Sorting probability graph.

A greater number of studies with larger sample sizes compared RNSM and CNSM (Figure 3A). The total operation time was significantly longer in both the ENSM group (WMD: 63.4; 95% CI: 21.18–105.59) and the RNSM group (WMD: 61.22; 95% CI: 24.26–98.24) compared to the CNSM group (Figure 3B). Based on total operation time, CNSM emerged as the most favorable surgical approach, followed by RNSM (Figure 3C).

#### Blood loss (mL)

**Figure 4.**
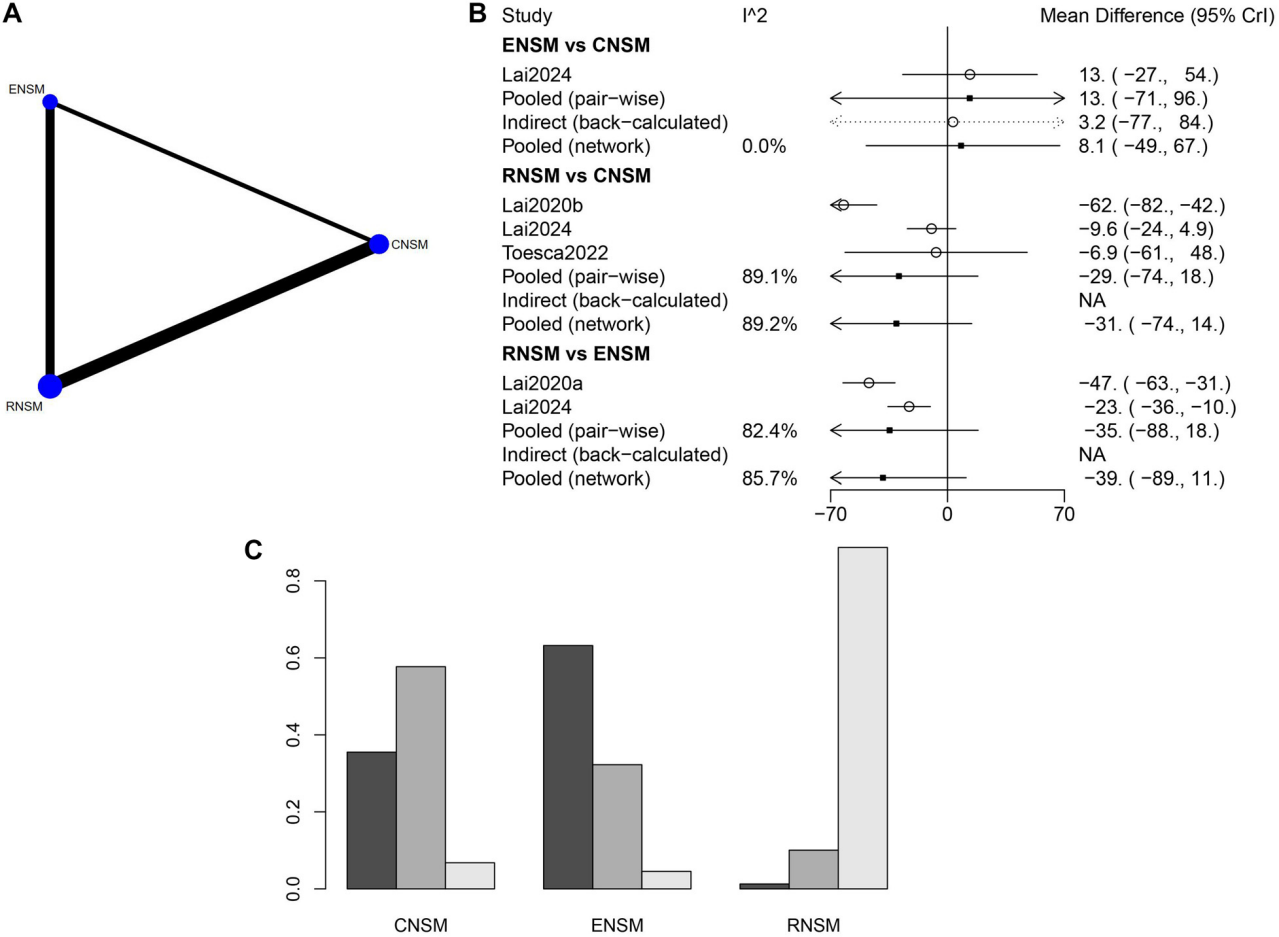
**Meta-analysis results of blood loss.** (A) Network diagram; (B) Forest plot; (C) Sorting probability graph.

A greater number of studies with larger sample sizes were available for the direct comparison between RNSM and CNSM (Figure 4A). The forest plot revealed no significant differences in intraoperative blood loss among the groups (Figure 4B). However, based on the ranking analysis, RNSM appeared to be the most favorable approach for minimizing blood loss, followed by CNSM (Figure 4C).

#### Hospital stay (days)

**Figure 5.**
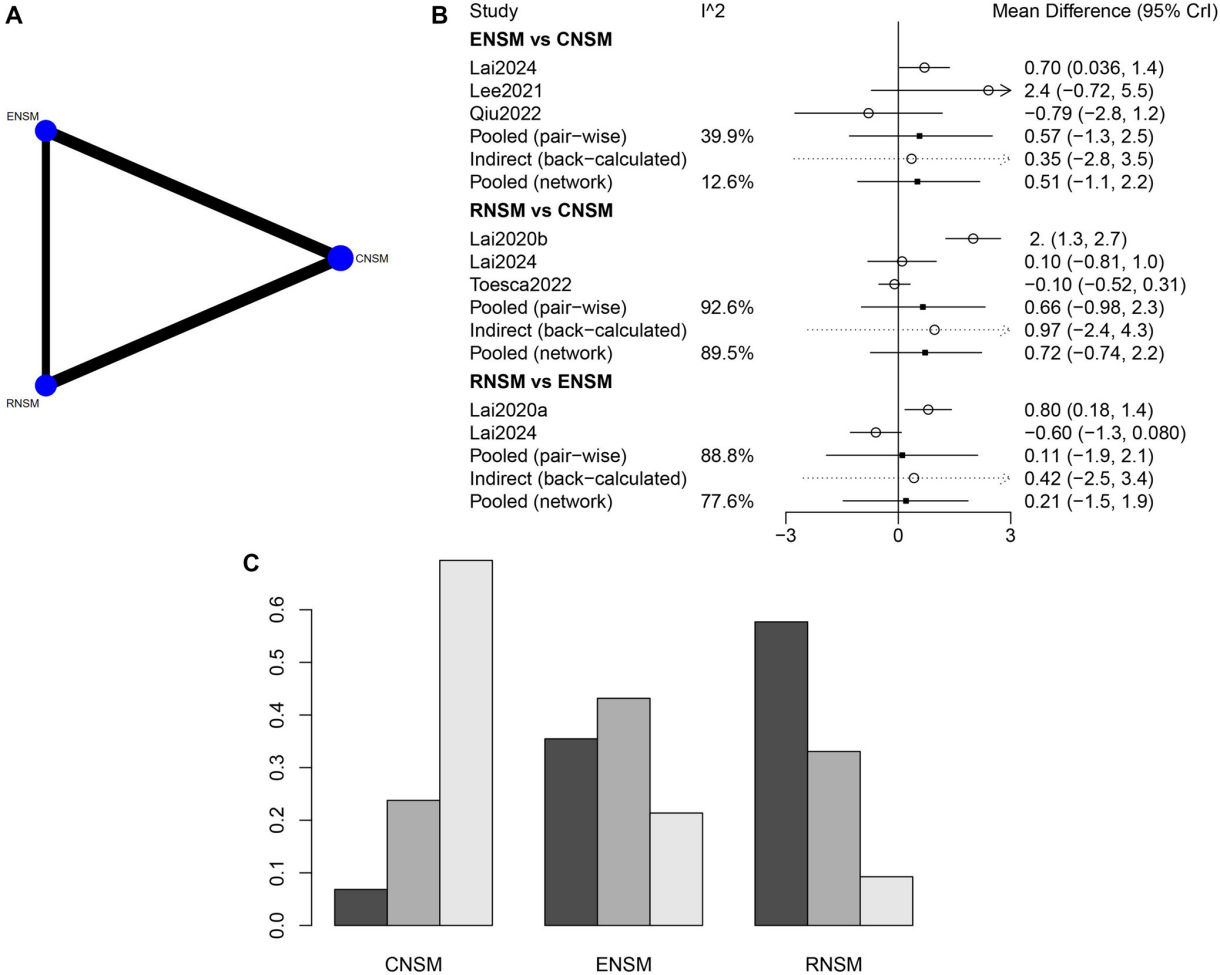
**Meta-analysis results of hospital stay meta.** (A) Network diagram; (B) Forest plot; (C) Sorting probability graph.

As illustrated in Figure 5A, larger sample sizes were observed in the comparisons between RNSM and CNSM, as well as between ENSM and CNSM. However, the forest plot demonstrated no statistically significant differences in hospital stay duration among the groups (Figure 5B). Based on the ranking analysis in Figure 5C, CNSM appeared to be the most favorable approach for reducing hospital stay, followed by ENSM.

#### Complications

**Figure 6.**
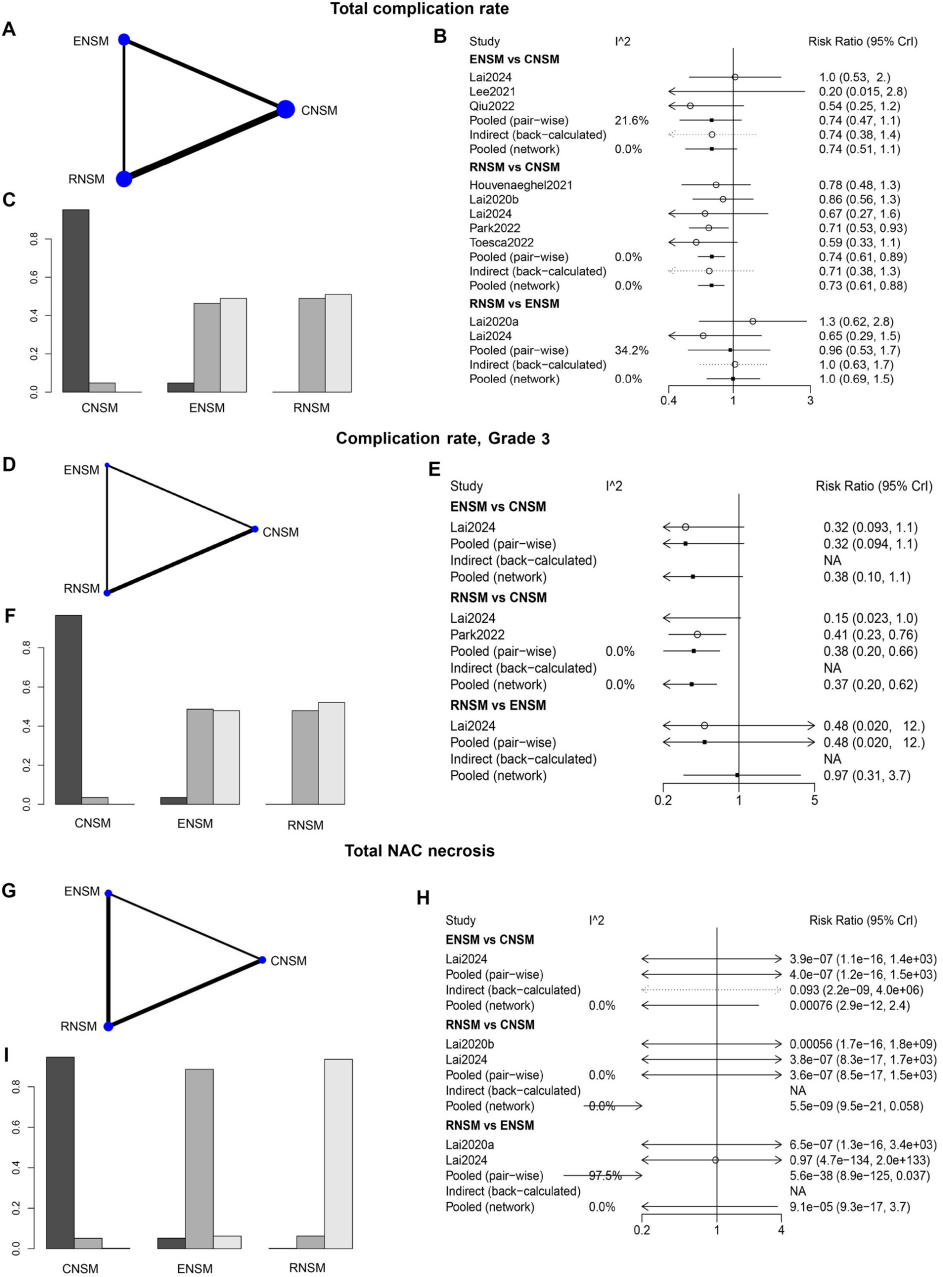
**Meta-analysis results of complication (total complication rate; complication rate, grade 3 and total nipple-areola complex [NAC] necrosis).** (A, D, and G) Network diagram; (B, E, and H) Forest plot; (C, F, and I) Sorting probability graph.

Overall, a greater number of studies with larger sample sizes were available for direct comparisons between RNSM and CNSM (Figure 6A, 6D, and 6G). Compared with CNSM, RNSM was associated with a significantly lower incidence of overall complications (WMD: 0.73; 95% CI: 0.61–0.88), grade 3 complications (WMD: 0.37; 95% CI: 0.20–0.62), and total NAC necrosis (WMD: 5.5e-09; 95% CI: 9.5e-21–0.058) (Figure 6B, 6E, and 6H). In the ranking analysis, RNSM had the lowest incidence of these complications, followed by ENSM, with CNSM showing the highest incidence (Figure 6C, 6G, and 6I). However, no significant differences were observed among the groups in other complications, including hematoma, infection, and implant loss (Figure S1).

#### Positive margin involvement

**Figure 7.**
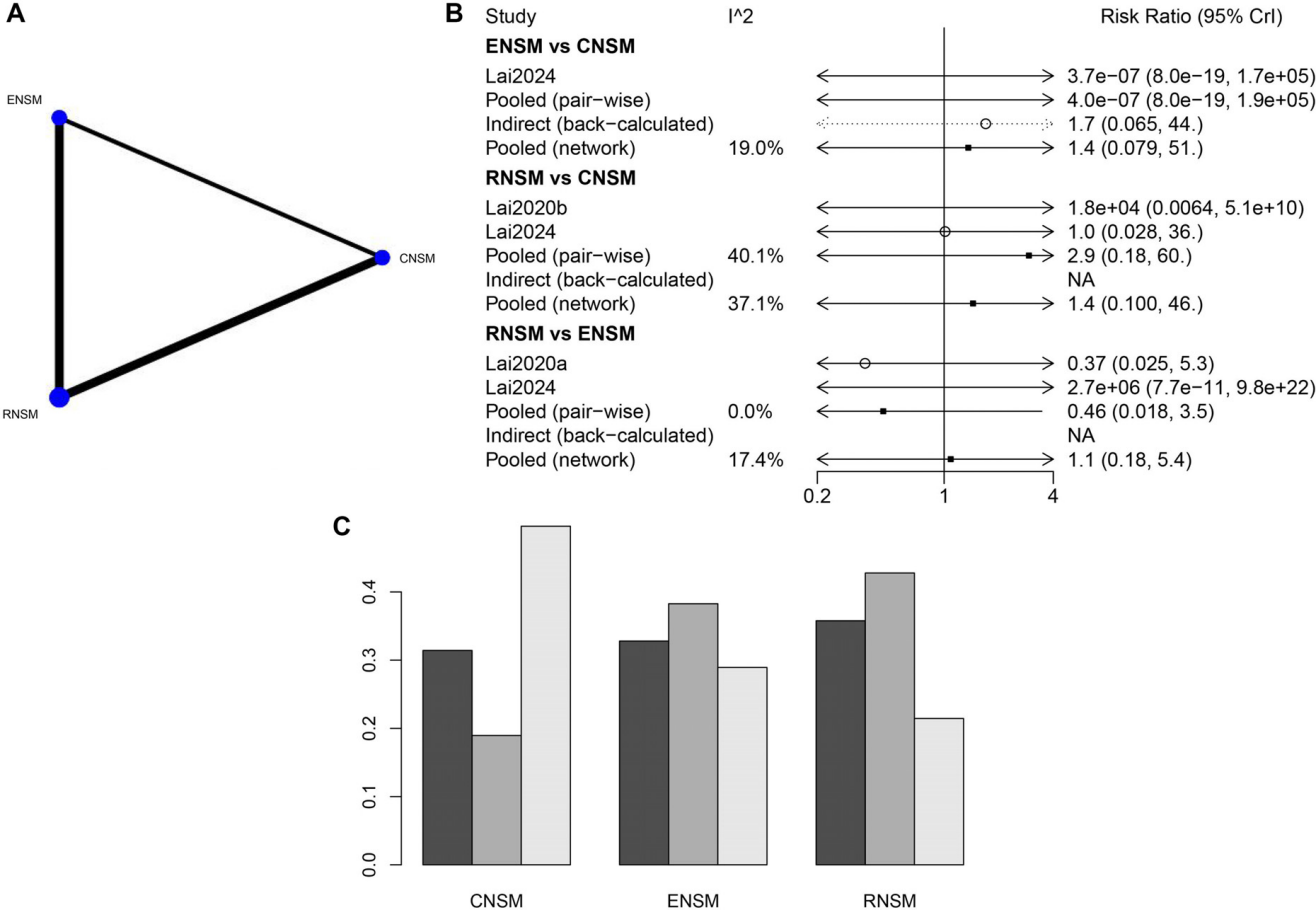
**Meta-analysis results of positive margin involvement.** (A) Network diagram; (B) Forest plot; (C) Sorting probability graph.

A substantial number of studies with large sample sizes were available for the direct comparison between RNSM and CNSM (Figure 7A). However, no statistically significant differences were observed between the groups regarding positive margin involvement (Figure 7B). Based on the ranking analysis, CNSM appeared to be the most favorable surgical approach for minimizing positive margin involvement (Figure 7C).

**Figure 8.**
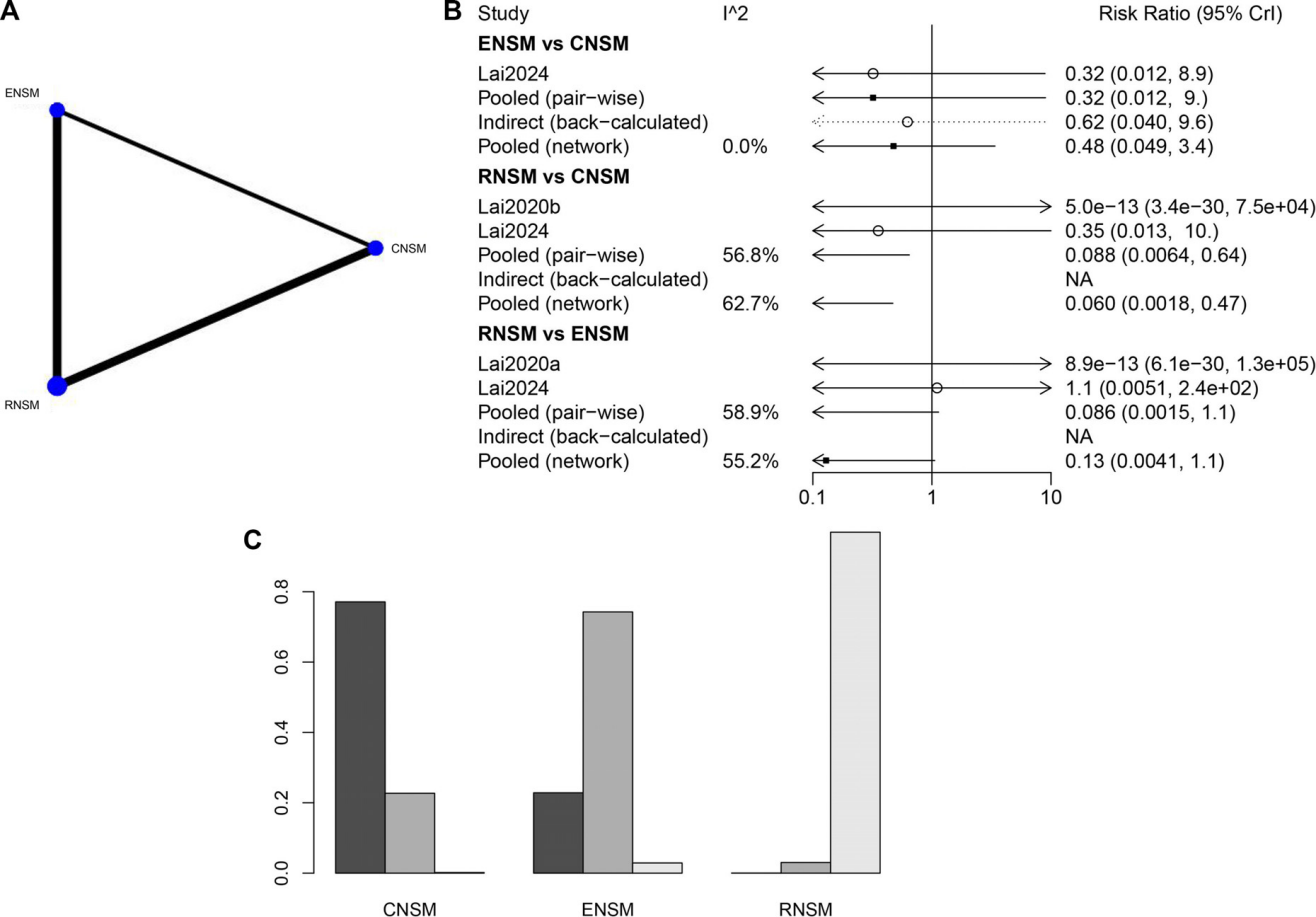
**Meta-analysis results of recurrence.** (A) Network diagram; (B) Forest plot; (C) Sorting probability graph.

#### Recurrence

A substantial number of studies with large sample sizes were available for the direct comparison between RNSM and CNSM (Figure 8A). As shown in Figure 8B, the recurrence rate was significantly lower in the RNSM group compared to the CNSM group (WMD: 0.060; 95% CI: 0.0018–0.47). According to the ranking probability graph (Figure 8C), RNSM was associated with the lowest recurrence rate, followed by ENSM, while CNSM had the highest recurrence rate.

## Discussion

Despite the increasing survival rates of patients with breast cancer following surgical treatment, a subset of patients continues to experience recurrence or metastasis [39–41]. In recent years, the clinical outcomes of RNSM and ENSM have been extensively studied. Both approaches have been shown to offer esthetic advantages, including scarless surgery and high patient satisfaction [42, 43]. However, the comparative efficacy of RNSM, CNSM, and ENSM when combined with IPBR remains uncertain. This NMA included 10 studies to evaluate the effectiveness of RNSM, CNSM, and ENSM combined with IPBR in the treatment of breast cancer. The risk of postoperative complications is influenced by both patient-related and surgery-related factors. While the mastectomy technique significantly impacts patient outcomes, the type of IPBR also plays a critical role. For example, submuscular IPBR is more commonly associated with postoperative pain, restricted shoulder mobility, and animation deformity, whereas prepectoral IPBR increases the risk of rippling. Among the included studies, Lai et al. [32] employed submuscular IPBR, Qiu et al. [36] primarily used submuscular IPBR but applied prepectoral IPBR in specific cases, and Moon et al. [34] utilized prepectoral IPBR. The remaining studies did not report the type of IPBR used [16, 17, 31, 33, 35, 37, 38]. As a result, a subgroup analysis based on IPBR technique was not feasible due to insufficient reporting. Of the 10 studies included, one originated from France [17], three from South Korea [33–35], one from Italy [37], and five from China [16, 31, 32, 36, 38]. Notably, European studies tended to be multicenter prospective cohorts, whereas studies from China and South Korea were predominantly single-center retrospective designs. This discrepancy may have led to an overestimation of the short-term benefits of RNSM, such as reduced hospital stay. Moreover, European research emphasized long-term survival outcomes, while Asian studies focused more on short-term efficacy, with limited reporting on long-term complications. Geographical differences and cultural practices may further contribute to technical variations in surgical procedures. Moving forward, international collaboration will be critical to harmonize evidence quality and address regional disparities. Such efforts are essential to support the global implementation of individualized treatment strategies for breast cancer.

Our meta-analysis found no statistically significant differences in clinical outcomes between RNSM and ENSM when combined with immediate implant-based breast reconstruction. The robotic surgical system has been shown to reduce the surgeon’s physical workload while enhancing procedural precision [35]. RNSM is increasingly favored due to its ability to facilitate more accurate and efficient breast tissue removal. It offers several advantages, including smaller incisions, reduced intraoperative bleeding, and a lower incidence of surgical complications—factors that contribute to improved postoperative quality of life [42]. Lee et al. [44] reported significantly lower rates of postoperative papillary necrosis and complications in the RNSM group compared to the CNSM group. Furthermore, RNSM combined with IPBR has been associated with a reduced risk of major necrosis [45]. In this NMA, RNSM with IPBR demonstrated superior outcomes compared to CNSM with IPBR, showing lower overall complication rates, fewer grade 3 complications, and a decreased incidence of total NAC necrosis. Additionally, the recurrence rate was lower in the RNSM + IPBR group than in the CNSM + IPBR group.

RNSM represents a novel surgical strategy for patients with breast cancer and has been associated with low perioperative morbidity, enhanced cosmetic outcomes, and better preservation of nipple sensitivity [46]. It has been widely reported as an effective and safe option for both treatment and prevention [47–49]. ENSM, as a minimally invasive approach, also provides favorable cosmetic results, inconspicuous scarring, and high levels of patient satisfaction [50]. A previous study demonstrated that both RNSM and ENSM were associated with improved wound healing compared to CNSM, albeit with higher associated costs [32]. Our findings showed that ENSM combined with IPBR resulted in significantly shorter incisions than CNSM. Furthermore, both RNSM and ENSM, when combined with IPBR, were superior to CNSM in terms of surgical incision length, complication rates, and recurrence outcomes—with RNSM + IPBR showing the most favorable results overall. However, CNSM combined with IPBR remained superior to both RNSM and ENSM approaches with respect to total operation time, length of hospital stay, and the incidence of positive margin involvement.

Several previous meta-analyses have evaluated the efficacy of RNSM compared to CNSM or ENSM in the surgical treatment of breast cancer. For instance, one meta-analysis comparing RNSM and CNSM reported that RNSM was associated with significantly longer operative times, a lower rate of necrosis, and fewer overall complications [51]. Another meta-analysis found no significant difference in complication rates between NSM and RNSM, suggesting that RNSM is a safe surgical option for patients undergoing mastectomy [52]. Additional studies have confirmed the feasibility of RNSM and its acceptable short-term efficacy [53]. Compared to CNSM, minimally invasive NSM techniques—such as RNSM and ENSM—are associated with longer operative and hospitalization times, but they offer benefits including reduced intraoperative blood loss, a lower incidence of complications and nipple necrosis, and significantly improved patient satisfaction [54]. These findings align with the results of the present study. Nonetheless, due to the higher costs and extended surgical duration associated with RNSM, its use may be best reserved for selected cases in which its advantages are most likely to yield substantial clinical benefit.

This meta-analysis presents several notable strengths. First, a comprehensive and systematic literature search strategy was employed to minimize the risk of publication bias. Second, the use of Bayesian statistical methods enabled the ranking of all included interventions, offering more precise and robust comparative estimates. Lastly, to the best of our knowledge, this is the first study to systematically evaluate and compare the clinical outcomes of different NSM techniques (CNSM, RNSM, and ENSM) when combined with IPBR.

Despite its strengths, this study has several limitations. First, some of the included studies had small sample sizes, which may have affected the robustness and stability of the results. Second, due to the limited number of available studies, subgroup analyses exploring the influence of different treatment strategies on clinical outcomes could not be conducted. Third, the absence of consistent reporting on outcome timing prevented stratification by specific follow-up periods. Therefore, while the findings highlight the potential advantages of RNSM and ENSM, further large-scale, independent studies with standardized reporting and long-term follow-up are necessary to validate and strengthen the conclusions of this meta-analysis.

## Conclusion

In summary, this NMA suggests that RNSM and ENSM combined with IPBR offer superior outcomes compared to CNSM combined with IPBR, particularly in terms of shorter surgical incisions, reduced complication rates, and lower recurrence. Overall, RNSM and ENSM combined with IPBR demonstrate greater efficacy and safety than conventional approaches. Nevertheless, high-quality randomized controlled trials are necessary to confirm and further substantiate these findings.

## Supplemental data

Supplemental data are available at the following link: https://www.bjbms.org/ojs/index.php/bjbms/article/view/11687/3840.

## Data Availability

Data can be obtained from corresponding authors.
